# Fecal Shedding of Multidrug Resistant *Escherichia coli* Isolates in Dogs Fed with Raw Meat-Based Diets in Brazil

**DOI:** 10.3390/antibiotics11040534

**Published:** 2022-04-17

**Authors:** Carolina Pantuzza Ramos, Carolina Yumi Iceri Kamei, Flávia Mello Viegas, Jonata de Melo Barbieri, João Luís Reis Cunha, Yaovi Mahuton Gildas Hounmanou, Fernanda Morcatti Coura, Jordana Almeida Santana, Francisco Carlos Faria Lobato, Anders Miki Bojesen, Rodrigo Otávio Silveira Silva

**Affiliations:** 1 Departamento de Medicina Veterinária Preventiva, Universidade Federal de Minas Gerais (UFMG), Belo Horizonte 30123-970, Brazil; carolina.pantuzza@gmail.com (C.P.R.); carolyumik@gmail.com (C.Y.I.K.); flaviaviegas95@gmail.com (F.M.V.); jonata_melobarbieri@hotmail.com (J.d.M.B.); jaumlrc@gmail.com (J.L.R.C.); jordanaalmeidasantana@gmail.com (J.A.S.); franciscolobato@vetufmg.edu.br (F.C.F.L.); 2 Department of Veterinary and Animal Sciences, Faculty of Health and Medical Sciences, University of Copenhagen, 1870 Copenhagen, Denmark; gil@sund.ku.dk (Y.M.G.H.); miki@sund.ku.dk (A.M.B.); 3 Departamento de Ciências Agrárias, Instituto Federal de Minas Gerais (IFMG), Bambuí 38900-000, Brazil; fernanda.coura@ifmg.edu.br

**Keywords:** RMBD, multidrug-resistant, Enterobacteriaceae

## Abstract

The practice of feeding dogs raw meat-based diets (RMBDs) is growing in several countries, and the risks associated with the ingestion of pathogenic and antimicrobial-resistant *Escherichia coli* in dogs fed these diets are largely unknown. We characterized *E. coli* strains isolated from dogs fed either an RMBD or a conventional dry feed, according to the phylogroup, virulence genes, and antimicrobial susceptibility profiles of the bacteria. Two hundred and sixteen *E. coli* strains were isolated. Dogs fed RMBDs shed *E. coli* strains from the phylogroup E more frequently and were positive for the *E. coli* heat-stable enterotoxin 1-encoding gene. Isolates from RMBD-fed dogs were also frequently positive for multidrug-resistant *E. coli* isolates including extended-spectrum beta-lactamase (ESBL) producers. Whole-genome sequencing of seven ESBL-producing *E. coli* strains revealed that they predominantly harbored *blaCTX-M-55*, and two strains were also positive for the colistin-resistant gene *mcr-1*. These results suggest that feeding an RMBD can affect the dog’s microbiota, change the frequency of certain phylogroups, and increase the shedding of diarrheagenic *E. coli*. Also, feeding an RMBD seemed to be linked with the fecal shedding of multidrug-resistant *E. coli*, including the spread of strains harboring mobilizable colistin resistance and ESBL genes. This finding is of concern for both animal and human health.

## 1. Introduction

In recent years, an increasing number of owners have been feeding their pets raw meat-based diets (RMBDs) [1,2,3,4,5]. However, several studies have demonstrated the pathologic risks associated with this practice due to the potential contamination of meats with zoonotic microorganisms and the subsequent risk of fecal shedding, which is a threat to animal and human health due to the potential environmental spread of pathogenic microorganisms [5,6,7,8]. Moreover, there is a strong association between feeding pets raw food and the shedding of extended-spectrum beta-lactamase (ESBL)-positive Enterobacteriaceae in household cats [9,10]. Interestingly, despite these findings and health agency statements regarding the risks, studies have shown that owners are either unaware of or tend to ignore the risks posed by feeding RMBDs [5,7]. Although increased shedding of important pathogens by dogs fed RMBDs has been demonstrated, only few studies have characterized the *E. coli* strains isolated from dogs fed this type of diet. Some studies have reported that an RMBD may influence the antimicrobial susceptibility patterns of Enterobacteriaceae, increasing the fecal shedding of multidrug-resistant and ESBL-positive strains [6,10,11,12]. However, the virulence factors present in these isolates remain less explored. In addition, a deep molecular characterization of these multiresistant isolates is lacking. It is important to highlight several studies demonstrating a possible exchange of pathogenic *E. coli* between infected humans and their healthy dogs, strongly suggesting the role of dogs as carriers of these zoonotic strains [13,14,15]. Several studies have demonstrated highly similar genotypes between isolates from dogs and their owners, supporting the zoonotic potential of these strains [13,14,15,16]. Considering the virulence potential of *E. coli*, as well as the possible animal and public health hazards associated with the emergence of antimicrobial-resistant strains [10,17] and the sharing of *E. coli* between people and pets in the household [13,14,16], the present study aimed to characterize and compare *E. coli* strains isolated from dogs fed either an RMBD or a conventional dry diet, based on the virulence genes, phylogroups, and antimicrobial resistance profiles of the bacteria.

## 2. Results

### 2.1. Phylogroups and Virulence Factors

A total of 212 *E. coli* strains were isolated from the feces of 92 dogs (Table S1). All phylogroups of *E. coli* were detected among the isolates, where B1 and B2 were the most common phylogroups detected (32.0 and 22.6%, respectively), and 8.4% (18/2126) of the strains were not assigned to any phylogroup (Table 1).

### 2.2. Antimicrobial Resistance

High rates of resistance to trimethoprim/sulfamethoxazole (44.3%), ampicillin (34.9%), and ciprofloxacin (34.9%) were detected. Additionally, low resistance to amoxicillin/clavulanic acid (3.7%), florfenicol (7.0%), and neomycin (1.8%) was detected (Table 2). *E. coli* isolates from dogs fed RMBDs were more frequently resistant to 9 out of the 11 tested antimicrobials (*p* < 0.01).

**Figure 1:**
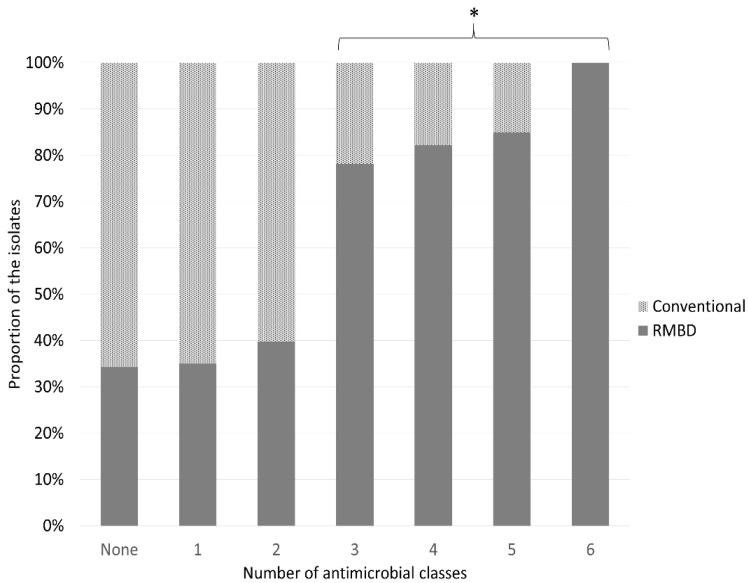
Percentages (%) of *E. coli* isolates from dogs fed raw meat-based diets (RMBDs) (*n* = 85) or conventional dry feed (*n* = 127) that are resistant to different numbers of antimicrobial classes. (*) Dogs fed an RMBD were more likely to shed *E. coli* strains resistant to three or more antimicrobials (95% CI: 3.6–14.7; *p* = 0.0004).

**Figure 2:**
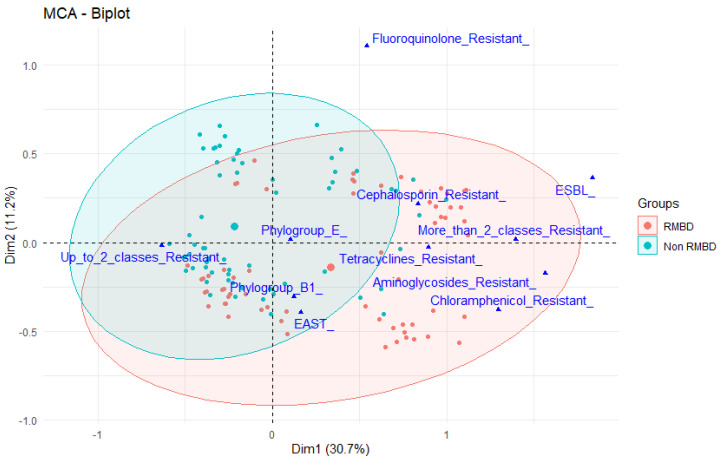
Multiple correspondence analysis (MCA) of categorical variables of *E. coli* from dogs fed raw meat-based diets (RMBDs) (red) or conventional dry feed (blue). This two-dimensional biplot graphic explains 41.9% of total variation and comprises 95% of *E. coli* isolates within the ellipse. Variables were considered to be associated when they plotted closely together.

*E. coli* from dogs fed an RMBD were more frequently multidrug-resistant (Figure 1) (*p* < 0.01). Fecal shedding of ESBL-producing *E. coli* strains was observed in six dogs (3.7% of *E. coli*). ESBL-producing *E. coli* were also more frequent among dogs fed RMBD (95% CI: 2.093–670.2; *p* = 0.001). Resistance to aminoglycosides and sulfonamide in isolates from RMBD-fed dogs was also evaluated using correspondence analysis and plotted closely to phylogroup E and EAST-1-positive strains (Figure 2).

**Figure 3:**
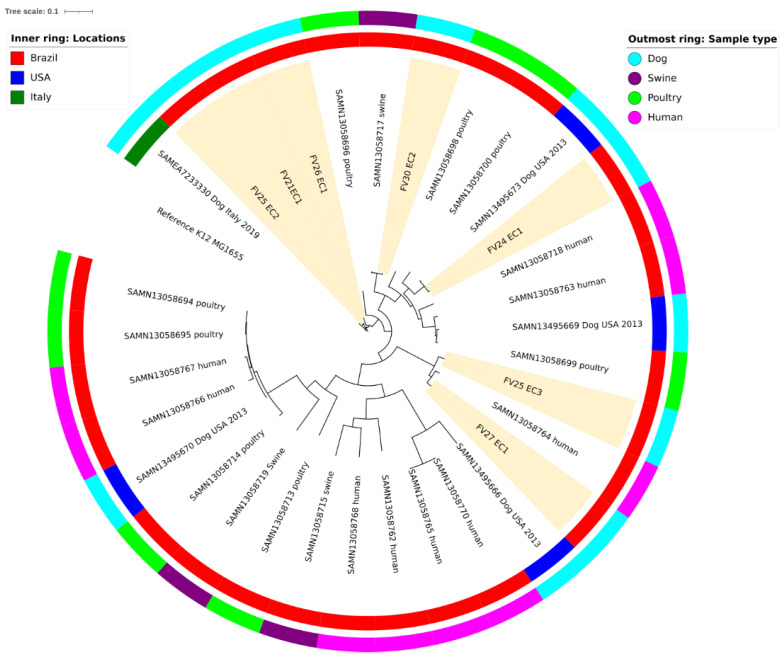
Phylogenetic tree of single-nucleotide polymorphisms (SNPs) found in the core and accessory genome from the seven *E. coli* isolated from dogs fed raw meat-based diets (RMBDs). Ten isolates from dogs, swine, poultry, and humans (outmost ring: sample type) from Brazil, Italy, and USA (inner ring: locations) were added for comparison purposes; cluster formation among RMBD-fed dogs (FV30 EC2, FV24 EC1, FV25 EC3, and FV27 EC1), swine, humans, and poultry *E. coli* are observed.

In the present study, ESBL-producing *E. coli* were identified only in dogs fed RMBD. Seven *E. coli* strains identified as ESBL producers were subjected to whole-genome sequencing (Table 3). *The blaCTX-M* genes were found in six isolates, *blaTEM* in three isolates, and *blaSHV* in one isolate. Two strains were also positive for the colistin-resistant gene *mcr-1*. The *blaCTX-M-55* gene is the most common blaESBL gene. Multilocus sequence typing (MLST) analysis of the isolates revealed strains classified as ST10 (*n* = 2), ST57 (*n* = 2), and ST410 (Table 3). Two strains classified as ST57 and ST410 were positive for the colistin-resistant gene *mcr-1*. A BLAST analysis of the nodes containing the ESBL and *mcr-1* genes revealed that they were all located in mobile genetic elements of variable replicon types, including the IncFII plasmid, found in all isolates, except one (Table 4). All these ESBL and *mcr-1* genes were located on contigs with a high sequence identity and query cover (98–100% identity) with the plasmids and other mobile genetic elements of *E. coli* strains isolated mostly from chickens, humans, and cattle (Table 4). In addition, a single nucleotide polymorphisms (SNP) analysis of the core and accessory genomes revealed phylogenetic clades composed of *E. coli* isolates from humans, poultry, swine, and ESBL-positive dogs from Brazil (Figure 3 and Table S2).

## 3. Discussion

In the present study, phylogroups B1 and B2 were the most common phylogroups detected, which was similar to previous reports of animal isolates, including dogs [19,20,21,22,23,24]. Interestingly, phylogroup B2 was more frequently isolated from dogs fed conventional dry feed, while phylogroup E was more commonly isolated from dogs fed RMBDs. Dietary habits are known to alter the composition and diversity of the intestinal microbiota, including *E. coli*, which may explain the differences among the lineages of *E. coli* detected here [25,26,27]. *E. coli* from phylogroup B2 are frequently isolated from various species of herbivorous and omnivorous mammals, including dogs [21,24,28]. The amounts of dietary fiber and carbohydrates are also known to strongly influence the composition of the gut microbiome [4,26,29] and modify the abundance of phylogroup B2 strains in the gut [26,30]. It is worth noting that RMBDs are characterized by their low carbohydrate content (approximately 15%), which is significantly lower than that of commercial dry diets [4]. Curiously, phylogroup E has not been frequently isolated from animal carcasses [23,31,32], although it may be associated with *E. coli* isolated from cattle [23].

Dogs fed RMBDs seemed to be more likely to shed *E. coli* isolates positive for the EAST-1 toxin-encoding gene. The role of *E. coli* as a cause of diarrhea in dogs is largely unknown [33,34]. However, there is strong evidence of the zoonotic potential of some pathotypes that are responsible for different clinical manifestations in humans [14,17,35]. Hence, similar to previous reports [13,33,35], fecal shedding of pathogenic *E. coli* by dogs suggests that these animals are potential reservoirs of pathogenic *E. coli*. EAST-1 positive strains have been associated with several outbreaks of diarrhea in humans [36,37,38]. This adds to the list of potential zoonotic pathogens, including *Salmonella* spp. and Enterobacteriaceae [1,5,30], shed in the feces of dogs fed RMBDs. Previous studies have demonstrated the presence of EAST-1-positive strains in the carcasses of food animals [37,39,40]. Thus, raw meat is one potential source of these strains, which suggests a hypothesis for the high level of shedding of these potential zoonotic agents by dogs fed RMBDs. Interestingly, these studies showed that these isolates were highly similar to those recovered from humans with diarrhea caused by EAST-1-positive strains, suggesting a possible zoonotic link.

In a multiple correspondence analysis (MCA), there was an association between phylogroup E and the presence of EAST-1-positive strains in dogs fed RMBDs (Figure 2). A recent study of *E. coli* isolates from diarrheic dogs showed that several belonged to phylogroup E [21]. Interestingly, a correlation has been reported between RMBDs and an increased risk of diarrhea in dogs [1,6]. This highlights the need for more studies on the role of *E. coli* as an etiological agent of enteric diseases and the influence of RMBDs in these cases.

In the present study, *E. coli* isolates from dogs fed RMBDs were more likely to be multidrug-resistant, and a dog fed an RMBD was approximately seven times more likely to shed a multidrug-resistant *E. coli* strain. These results corroborate those of previous studies showing that dogs are relevant reservoirs of multidrug-resistant bacteria [10,41]. Moreover, the association between the consumption of an RMBD and increased shedding of multidrug-resistant isolates is similar to the findings of other studies [6,11,12,27]. This is of great concern, especially for *E. coli*, because of the evidence of cross-species transmission of this bacterium [16,35,42]. Interestingly, the consumption of an RMBD increases the chance of *E. coli* transmission between owners and their dogs [10,42].

Data from the World Health Organization (WHO) estimate that antimicrobial resistance is responsible for at least 700,000 deaths per year worldwide [43]. Enterobacteriaceae resistant to third- and fourth-generation cephalosporins, including *E. coli*, are one of the most relevant pathogens and represent an increasing threat to public health [12,44,45]. In the present study, RMBD-fed dogs seemed to be more likely to shed *E. coli* strains resistant to third-generation cephalosporins. The higher rate of resistance to enrofloxacin in dogs fed RMBDs must also be highlighted because fluoroquinolones are commonly used in human and veterinary medicine and are classified as “critically important antimicrobials” by both the WHO and the World Organization for Animal Health [45,46]. Fecal shedding of strains resistant to aminoglycosides and aminopenicillins, which are also listed as critically important antimicrobials [45,46], was also higher in dogs fed RMBDs. Interestingly, resistance to some compounds was similar in the phylogroup E and EAST-1-positive strains (Figure 2). On the other hand, phylogroup B2 strains were not associated with resistance to any antimicrobial class, similar to previous studies that suggested a lesser tendency of B2 strains to express resistance determinants [24,47,48].

The presence of ESBL-*E. coli* in dogs and cats is a global phenomenon and is of concern due to the possibility of its spread to humans, wherein contact with pets is considered a risk factor for colonization, as previously described [49,50,51]. There are a few reports of ESBL-positive *E. coli* in healthy dogs from Brazil, with a frequency of 6.1–28.6% [49,52,53,54]. In the present study, feeding dogs with RMBDs alone was associated with fecal shedding of *E. coli* ESBL strains, corresponding to 15.7% of the animals. CTX-M-55 is the most common ESBL, which has been increasingly reported in companion animals [49,50,55,56,57] and has also been reported to cause infection in humans [58,59]. CTX-M-55 is a derivative of the widely distributed CTX-M-15 [60], and the decreasing occurrence of CTX-M-15 beta-lactamase producers over the last few years in favor of CTX-M-55 has been demonstrated [61]. The emergence of CTX-M-55 in dogs and cats in different countries around the world may indicate the spread of this enzyme because of international food or animal trade [62]. CTX-M-55 has been reported as one of the most common ESBL-producing *E. coli* found in food animals, including poultry [63,64,65,66], which was the main source of meat for all dogs in the present study. Recently, a study evaluating commercially available raw pet food in Switzerland found that more than 60% of the products had ESBL-producing Enterobacteriaceae. These strains commonly have bla_ESBL_ genes identical to those causing diseases in animals and humans worldwide [67], which emphasizes the risks that this feed poses to pets and owners.

The present study revealed three important sequence types among the seven ESBL-positive isolates recovered from dogs (ST10, ST57, and ST410). These sequence types have been previously reported in dogs, but also in food isolates, livestock, and, more importantly, in humans with bloodstream and urinary infections in several countries, including Brazil [10,50,55,56,57,61,62,68,69,70,71,72,73,74,75,76,77,78]. Studies have also reported strong evidence for clonal dissemination and interspecies transmission of ST410 and ST10, which have been associated as emerging and clinically relevant multidrug-resistant strains [50,68,74,78,79,80,81]. Two strains, classified as ST57 and ST410, were also positive for the colistin-resistant gene *mcr-1*, a critical resistance determinant found for the first time in *E. coli* from companion animals in Brazil. Those ESBL-producing *E. coli* sequence types carrying *mcr-1* have been reported in infections in humans worldwide [76,77,78,82,83]. In some of these reports, the authors also suggested that animals are the source of infection [78,82,83]. This finding is of concern as colistin is considered a last-resort antibiotic for human infections caused by multidrug-resistant Gram-negative pathogens, including ESBL strains [40,81,84], and its use has been banned in Brazil since 2016 [85].

To better understand the possible origin of the critically relevant strains, nodes containing the sequences of ESBL enzymes and *mcr-1* genes were subjected to a BLAST analysis. These genes were all located in mobile genetic elements with a high identity to *E. coli* isolated from sources other than dogs, including poultry, humans, and cattle (Table 4 and Figure S1). Interestingly, from all the identified replicons, IncFII was found in all isolates, except one. IncFII is widespread among the Enterobacteriaceae and is particularly successful in its ability to spread multidrug resistance and ESBL determinants among strains from several hosts [40,47,68,86,87]. In addition, an SNP analysis suggested a high genetic similarity among four ESBL-positive strains (FV30 EC2, FV24 EC1, FV25 EC3, and FV27 EC1) and isolates from swine, poultry, and humans (Figure 3). Unfortunately, genomes of dog *E. coli* isolated from Brazil were not available for comparison. It is possible to hypothesize that the *E. coli* present in dog microbiota acquired resistance determinants via horizontal exchange or that critical strains were acquired from a common ancestor, likely from their feed or via contact with different hosts [5,50,88,89].

It is important to note that the dogs included in this study did not undergo antimicrobial therapy during sampling. This is relevant since it is known that the use of antimicrobial drugs can increase the prevalence of resistant bacteria, including ESBL-producing *E. coli* [50]. Thus, it can be inferred that the fecal shedding of multidrug-resistant *E. coli* may be linked to the inclusion of raw products in the diet of dogs. Several studies have detected antimicrobial-resistant *E. coli* in raw meat destined for both human and animal consumption [8,37,39,41,90], and the prevalence of ESBL-producing *E. coli* is known to be high in chicken meat [91]. The use of antibiotics for promoting growth and treating diseases in food-producing animals is known to contribute to the spread of resistant bacteria through the food chain [92,93,94].

Since dogs from both groups were not in a controlled environment during this study, the influence of other factors related to the dog’s lifestyle could not be excluded, thus being a study limitation. The use of other drugs that can alter the microbiota, including proton-pump inhibitors and laxatives, was not evaluated, which is another limitation of this report [95]. Although a study using controlled dogs could provide unequivocal proof of the link between RMBD feeds and MDR bacteria, it is important to note that these results are in line with previously conducted studies on this subject.

## 4. Materials and Methods

### 4.1. Sampling

Healthy dogs fed an RMBD or a conventional dry feed diet were sampled in Minas Gerais, southeastern Brazil. Fecal samples were obtained from 38 dogs fed an RMBD and 54 dogs fed a conventional dry diet between December 2018 and July 2019 (Table S1) after the owners signed an informed consent term [5]. Only one dog per household was included; only animals that had not undergone antimicrobial therapy in the last 6 months were included in this study. All samples were collected immediately after evacuation, and only fecal material that did not come in contact with the floor was collected. The fecal material was stored in a cooler with ice packs, transported, and processed within 24 h. This study was approved by the Ethical Committee on Animal Use (CEUA-UFMG) under protocol 51/2015.

### 4.2. Isolation and Characterization of Escherichia coli Strains

To isolate *E. coli*, fecal samples were plated on MacConkey agar (Difco, Franklin Lakes, USA) plates and incubated for 24 h at 37 °C. Up to three lactose-fermenting colonies selected from each sample were subjected to species-specific polymerase chain reaction (PCR) [96]. To increase the chances of obtaining different clones from the same animal, isolates were chosen based on their morphological differences. *E. coli* strains were then classified into one of the different phylogroups (A, B1, B2, C, D, E, F, or Clade I) according to the presence or absence of the genes *chuA*, *arpA*, *and yjaA* as well as the DNA fragment TspE4.C2 [18,97]. The virulence genes associated with enterotoxigenic *E. coli* (ETEC; *sta*, *stb*, *lt*, *f5*, *f18*, *f41*, *f4*, and *987p*), enteropathogenic *E. coli* (EPEC; *eae*, *bfpA*, *iha*, *toxB*, and *efa1*), Shiga toxin-producing *E. coli* (STEC; *stx1*, *stx2*, *ehxA*, and *saa*), enterohemorrhagic *E. coli* (EHEC; *eae*, *iha*, *toxB*, *efa1*, *stx1*, *stx2*, *ehxA*, and *saa*), necrotoxigenic *E. coli* (NTEC; *cnf1*, *cnf2*, and *f17*), enteroaggregative *E. coli* (EAEC; *astA*, *aggR*, *aaf*, and *pet*), enteroinvasive *E. coli* (EIEC; *ipaH*), and EAST-1 genes were detected by PCR as previously described [19]. The reference strains EDL 933 (*eaeA*, *stx1*, *stx2*, *ehxA*, *iha*, *toxB*, and *efa1*), B41 (*f41*, *f5*, and *sta*), EAEC O42 (*astA*, *aggR*, *aaf*, and *pet*), S5 (*f17* and *cnf2*), NTEC1 (*cnf1*), STECLBA05 (*saa*), EIEC (*ipaH*), 2568 (*stb*, *f18*, and *stx2e*), 2569 (*lt* and *k88*), 2570 (*987p*), and E2348/69 (*bfpA*) were used as positive controls.

### 4.3. Antimicrobial Susceptibility

The antimicrobial susceptibility of *E. coli* strains was evaluated using the disc diffusion method according to the Clinical and Laboratory Standards Institute (CLSI) and the European Committee on Antimicrobial Susceptibility Testing (EUCAST) guidelines [98,99]. The inhibition zones were interpreted for seven different antimicrobial classes using the following representative drugs: trimethoprim/sulfamethoxazole (25 µg), enrofloxacin (5 µg), gentamicin (10 µg), neomycin (30 μg), ceftiofur (30 μg), amoxicillin/clavulanic acid (30 µg), ampicillin (10 μg), florfenicol (30 μg), doxycycline (30 μg), oxytetracycline (30 µg) (clinical breakpoints interpreted according to CLST [98]), and ciprofloxacin (5 µg), with the breakpoints interpreted according to the EUCAST [99] (DME, Araçatuba, Brazil). The *E. coli* reference strain ATCC 25922 was included as a control. Strains resistant to three or more antimicrobial classes were classified as multidrug-resistant, as recommended in previous studies [19,100]. The ETEST^®^ ESBL (TZ/TZL) strips (BioMérieux, Marcy-l’Étoile, France) were used to detect ESBL strains. The test was performed according to the manufacturer’s instructions. Briefly, *E. coli* strains were plated onto Mueller–Hinton agar, on which the E-test ESBL strip was placed on the center of the plate. The plates were incubated aerobically at 37 °C for 18 h. ESBL was detected as the presence of deformation of the TZ inhibition ellipse or a rounded phantom inhibition zone below the CT in the E-test strip edge.

### 4.4. Whole-Genome Sequencing Analysis

Seven ESBL-positive *E. coli* strains were subjected to whole-genome sequencing. Genomic DNA was extracted using the Maxwell 16^®^ Research Instrument (Promega, Madison, USA) combined with isozyme (10 mg/mL) and proteinase K (20 mg/mL). Genome sequencing was performed using the Illumina NextSeq platform (mid-out 2 × 150 bp cycles). The quality of the raw data was analyzed using FastQC (Babraham Bioinformatics), and the assembly was performed using SPAdes 3.5.0 [101]. Automatic annotation was performed using Prokka 1.10 (Rapid Bacterial Genome Annotation) software [102] with default parameters. ResFinder 4.1, PlasmidFinder 2.1, and VirulenceFinder 2.0 [103,104,105,106,107] were used to identify acquired antimicrobial resistance determinants and conjugative plasmid replicons. The nodes where critically important antimicrobial-resistant genes were located were subjected to BLAST analysis (https://blast.ncbi.nlm.nih.gov/Blast.cgi, accessed on 11 April 2022). MLST 2.0 was used to determine sequencing types according to the Achtman MLST scheme [105,108,109,110]. MLST analysis of *E. coli* isolates was performed using MLST 2.18.0 [110]. The core genome MLST of the seven isolates was performed using Ridom SeqSphere+ 4.1.9 [111]. Ten *E. coli* strains from previous studies on humans, swine, poultry, and dogs from the USA, Italy, and Brazil were also included for comparison purposes. The resistance genes and plasmid types were determined based on the CGE server [112], and the plasmid circle map was illustrated with BLAST Atlas using the GView server (https://server.gview.ca/,  accessed 20 December 2022) [113]. In all second-generation genome annotation files, contigs harboring the *blaCTX–M–55* gene were analyzed, and the *blaCTX–M–55* gene locations were roughly determined using BLAST.1. The seven *E. coli* genomes were phylogenetically analyzed with selected publicly available genomes of *E. coli* isolated from dogs, poultry, swine, and humans from Brazil, USA, and Italy (Table S2). A pool of 32 strains, containing our strains and public genomes, was subjected to SNP analysis using CSIPhylogeny [114] using *E. coli* K12 (MG1655) as a reference.

### 4.5. Statistical Analysiss

Isolates from the same animal and with the same phenotypic and genotypic profile were considered a single strain. The association of diet type (RMBD or conventional dry feed diet) and the pathovars, phylogroups, and antimicrobial susceptibility profiles of the *E. coli* isolates were assessed using R software (R Development Core Team, Wellington, New Zealand). Univariate analysis was performed using the Chi-square test and Fisher’s exact test, and variables with a *p*-value ≤ 0.2 were selected for multivariate analysis [115]. Selected variables were subjected to multivariate logistic regression by forward process modeling, and results with a *p*-value ≤ 0.05 were considered to be significant [116]. Odds ratios (ORs) and 95% confidence intervals (95% CIs) were calculated. MCA was performed in a two-dimensional graph using the same software, and variables were considered to be associated when they were plotted closely together [117].

## 5. Conclusions

In conclusion, these results suggest that RMBDs can change the *E. coli* composition in the canine gut microbiome, altering the frequency of certain phylogroups and increasing the shedding of diarrheagenic pathotypes. Additionally, our results suggest that RMBD intake increases the fecal shedding of multidrug-resistant *E. coli*, including ESBL and *mcr-1* strains, in dogs. This hypothesis should be further confirmed once it poses a potential risk not only for the dogs themselves but also to other animals and humans in proximity, due to the risk of spreading these bacteria both within the household and in the community.

## Figures and Tables

**Table 1:** Phylogroups of *E. coli* isolates from dogs fed with raw meat-based diets (RMBDs) and conventional dry feed. Different letters in a phylogroup column indicate statistical differences among dog groups fed with the different diets (*p* ≤ 0.05).

Type of Diet	Phylogenetic Groups (% Total)									Total
	A	B1	B2	C	D	E	F	Clade I	Unassignable ^¹^	
RMBD	6 (2.8)	31 (14.6)	9 (4.2) ^a^	10 (4.7)	0	15 (7.0) ^a^	7 (3.3)	0	7 (3.3)	85 (40.0)
Conventional	3 (1.4)	37 (17.4)	39 (18.3) ^b^	15 (7.0)	1 (0.4)	8 (3.7) ^b^	10 (4.7)	3 (1.4)	11 (5.1)	127 (59.9)
Total	9 (4.2)	68 (32.0)	48 (22.6)	25 (11.7)	1 (0.4)	23 (10.8)	17 (8.1)	3 (1.4)	18 (8.4)	212 (100)

¹ Identified as *E. coli* but not corresponding to any of the phylogroups according to Clermont et al. (2013) [18]. Phylogroup B2 was more frequently isolated from dogs fed conventional dry feed (95% CI: 0.1–0.56; *p* = 0.0002), while phylogroup E was more commonly isolated from dogs fed RMBDs (95% CI: 1.23–9.39; *p* = 0.01). *E. coli* isolates positive for the EAST-1 toxin-encoding gene were approximately 2.7 times more frequent in dogs fed RMBDs (95% CI: 1.11–7.29; *p* = 0.02).

**Table 2:** Frequencies (%) and *p* value of resistance to each tested antimicrobial drug in the *E. coli* strains isolated from dogs fed with raw meat-based diets (RMBDs) and conventional food.

Antimicrobial Drug	Type of Diet (% Total)		*p* Value
	RMBD (*n* = 85)	Conventional (*n* = 127)	
amoxicillin/clavulanic acid	5 (5.8)	3 (2.3)	0.2
ampicillin *	46 (54.1)	28 (22.0)	0.0004
ceftiofur *	30 (35.2)	25 (19.6)	0.01
enrofloxacin *	20 (23.5)	10 (7.8)	0.002
ciprofloxacin	29 (34.1)	45 (35.4)	0.7
trimethoprim/sulfamethoxazole *	52 (61.1)	44 (33.5)	0.0004
doxycycline *	31 (36.4)	22 (17.3)	0.003
oxytetracycline *	41 (48.2)	30 (23.6)	0.0001
florfenicol	9 (10.5)	6 (4.7)	0.09
gentamicin *	15 (17.6)	2 (1.5)	0.0004
neomycin *	4 (4.7)	0 (0)	0.03

* Statistical differences among dog groups fed with the different diets (*p* ≤ 0.05).

**Table 3:** Results of virulence factors and resistance gene detection and multilocus sequence typing (MLST) of the seven extended-spectrum beta-lactamase (ESBL)-positive *E. coli* isolates from six dogs fed raw meat-based diets in Brazil.

Animal	Isolate	MLST ^1^	Antimicrobial Resistance Genes		Virulence Factors
			ESBL ^2^	Other	
FV21	1	ST10	*bla*_CTX-M-55_	aph(3’)-Ia aadA22 mdf(A) lnu(F) gyrA* sul3 floR aadA22	*cif cma cvaC eae espA espB espF hlyF iucC iutA nleB ompT sitA tccP terC tir traT*
FV24	1	ST224	*bla*_CTX-M-55_ *bla*_TEM-1B_	gyrA* fosA3 mdf(A)	*cma cvaC gad hlyF iroN iss lpfA ompT sitA terC traT*
FV25	2	ST10	*bla*_CTX-M-55_	*aph(3′)-Ia mdf(A) mdf(A) aadA22 lnu(F)*	*cif cma cvaC eae espA espB gad hlyF iucC iutA ompTb sitA terC tir traT*
	3	ST57	*bla*_CTX-M-55_ *bla*_CTX-M-2_	aph(3′)-Ia sul1 dfrA7 mdf(A) floR gyrA* sul3 mdf(A) aadA1 mcr-1.1 fosA3 tet(A)	*astA cea chuA gad hra iha iss iucC iutA ompT sitA terC traT*
FV26	1	ST744	*bla*_CTX-M-55_ *bla*_TEM-1B_	*aph(3′)-Ia sul1 catA1 gyrA dfrA17 fosA3 aph(3′)-Ia mph(A) aadA5 tet(B) aph(6)-Id*	*terC traT*
FV27	1	ST57	*bla*_CTX-M-2_	ant(2″)-Ia sul2 dfrA1 mdf(A) aadA1 aadA1 gyrA*	*chuA cma etsC fyuA gad hlyF hra iroN irp2 iss iucC iutA ompT sitA terC traT tsh*
FV30	2	ST410	*bla*_SHV-12_ *bla*_TEM-1B_	aac(3)-Iid sul1 dfrA1 mdf(A) aadA1 gyrA* mcr-1.1	*astA cib cma cvaC etsC hlyF hra iroN iss iucC iutA lpfA ompT papC sitA terC traT*

^1^ Multilocus Sequence Typing (MLST)—Achtman scheme; ^2^ Extended-spectrum beta-lactamases (ESBL); * Resistance-associated mutations in *gyrA* gene.

**Table 4:** Detection of the conjugative plasmid replicons and similarity analyses of the critical important AMR genes detected in seven ESBL-positive *Escherichia coli* isolates from six dogs fed raw meat-based diets in Brazil.

Animal	Isolate	Relevant AMR Genes	Contig	Closest BLAST ^1^ Match Source, Country	Conjugative Plasmid Replicons
FV21	1	*bla*_CTX-M-55_	64	*E. coli* plasmid pRHB02-C09_2 (CP058073) Pig, UK	IncFIB; IncFIC; IncFII
FV24	1	*bla*_CTX-M-55_	168	*E. coli* plasmid pAH01-3 (CP055254) Poultry, China	IncFIB; IncFII; IncFII (pRSB107)
		*bla*_TEM-1B_			
FV25	2	*bla*_CTX-M-55_	70	*E. coli* plasmid pTREC1 (MN158989) Wetland sediment, USA	IncFIB; IncFIC (FII); IncFII; IncI2
	3	*bla*_CTX-M-55_	429	*E. coli* plasmid pAH01-3 (CP055254) Chicken, China	Col (MG828); Col156; IncFIB; IncFII; IncHI2; IncHI2A; IncI2; IncY
		*bla*_CTX-M-2_	74	*E. coli* Integron in117 (DQ125241) Human, Spain	
		*mcr-1*	334	*E. coli mcr-1* cassette (LT159973) Cattle, Germany.	
FV26	1	*bla*_CTX-M-55_	87	*Proteus mirabilis* genomic island PGI2C55 (MK847915) Chicken, China	IncFII; IncN; IncQ1
		*bla*_TEM-1B_			
FV27	1	*bla*_CTX-M-2_	284	*E. coli* plasmid RCS78_p (LT985296) Human, Brazil	ColpVC; IncFIB; IncFIC; IncI2
FV30	2	*bla*_SHV-12_	236	*E. coli* plasmid pMCR_1525_C2 (MT929281) Turkey, Brazil	ColpVC; IncFIA; IncFIB; IncFII; IncI1-I; IncX4
		*bla*_TEM-1B_	155	*E. coli* plasmid pSHE-CTX-M (CP022359) Human, France	
		*mcr-1*	183	*E. coli* plasmid pIncFIB_IncFII (CP066837) Chicken, USA	

**^1^** BLAST—Basic Local Alignment Search Tool (https://blast.ncbi.nlm.nih.gov/Blast.cgi, accessed on 11 April 2022).

## Data Availability

The data generated during the current study are available in the supplementary material. Publicly available datasets were analyzed in this study. This data can be found in www.ncbi.nlm.nih.gov, accessed on 11 Abril 2023 with the following accession numbers: CP055254; CP058073; MN158989; DQ125241; LT159973; MK847915; LT985296; MT929281; CP022359; CP066837; SAMN13058694; SAMN13058695; SAMN13058767; SAMN13058766; SAMN13058714; SAMN13058719; SAMN13058713; SAMN13058725; SAMN13058768; SAMN13058762; SAMN13058765; SAMN13058770; SAMN13058764; SAMN13058699; SAMN13058763; SAMN13058718; SAMN13058700; SAMN13058698; SAMN13058717; SAMN13058696; SAMN13495670; SAMN13495669; SAMN13495666; SAMN13495673; SAMEA7233330; and 7628977.

## Supplementary Materials

The following supporting information can be downloaded at: https://www.mdpi.com/article/10.3390/antibiotics11040534/s1, Figure S1. Location of the ESBL genes in the seven *E. coli* strains isolated from dogs fed RMBD around the poultry *E. coli* plasmid (accession number CP055254); Table S1. Raw data results—Phylogroups, virulence factors, and resistance genes; Table S2. Raw data results—SNP Analysis.
