# Supplementary material for: Fecal Shedding of Multidrug Resistant Escherichia coli Isolates in Dogs Fed with Raw Meat-Based Diets in Brazil
Source: Antibiotics (Basel). 2022 Apr 17;11(4):534. doi: 10.3390/antibiotics11040534 (PMC9029118; doi:10.3390/antibiotics11040534)
Supplement: Supplementary file 1 [file antibiotics-11-00534-s001.zip › Figure S1.pdf]

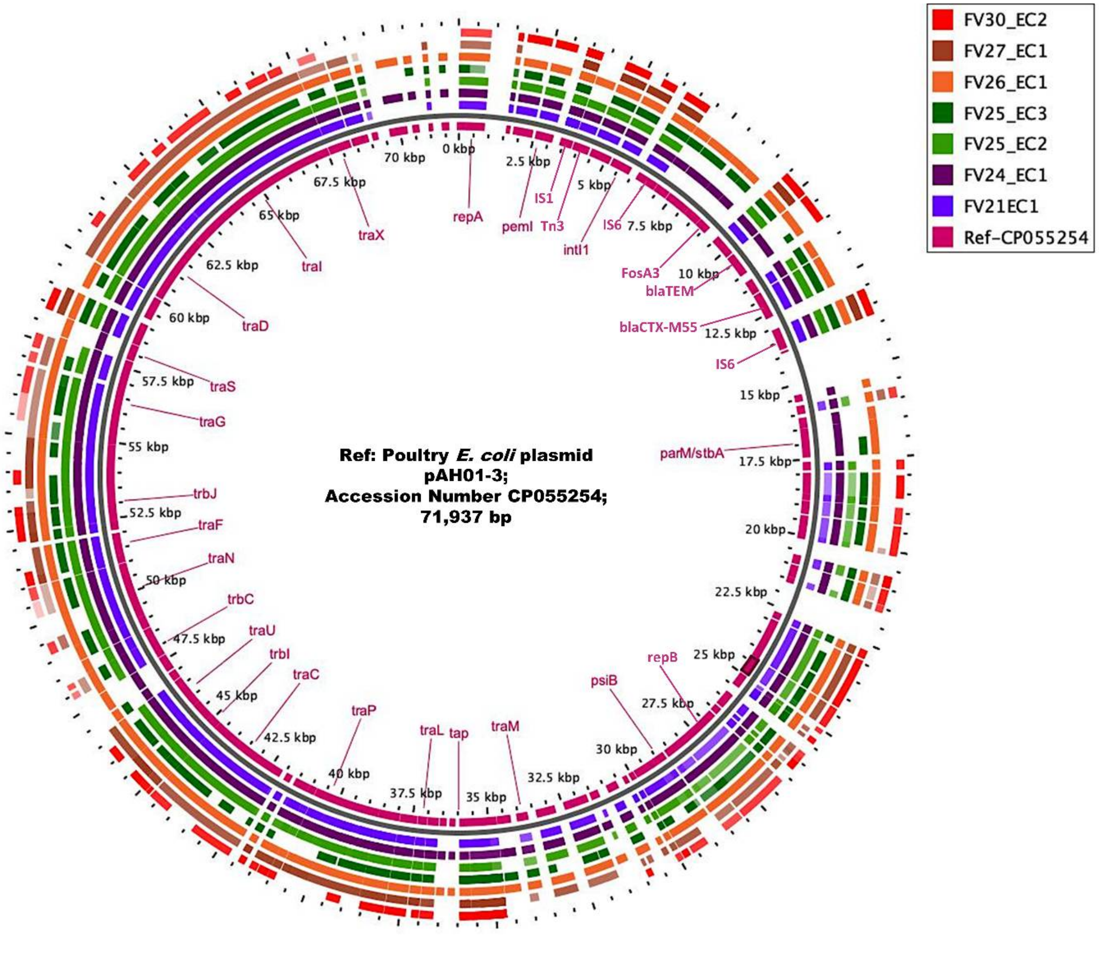

Figure S1. Location of the ESBL genes in the seven *E. coli* strains isolated from dogs fed RMBD around the poultry *E. coli* plasmid (accession number CP055254).
